# Understanding the transition to motherhood among adolescent mothers in rural Pakistan: Implications for mental health

**DOI:** 10.1371/journal.pone.0355175

**Published:** 2026-07-30

**Authors:** Amber Hussain, Tanya Park, Zahid Ali Memon, Salima Meherali

**Affiliations:** 1 Faculty of Nursing, University of Alberta, Edmonton, Alberta, Canada; 2 College of Healthcare Sciences, Nursing and Midwifery, James Cook University, Cairns, Queensland, Australia; 3 Department of Community Health Sciences, Aga Khan University, Karachi, Pakistan; Independent Global Health Consultant, INDIA

## Abstract

**Purpose:**

This study explored how adolescent mothers in Pakistan experience the transition to motherhood and the mental health challenges that arise during this period.

**Methods:**

A focused ethnographic study was conducted with 25 adolescent mothers in Matiari, Sindh. Data were collected through semi‑structured interviews, participant observations, and artifact elicitation. Meleis’ Transition Theory informed the study focus and development of the interview guide. Reflexive thematic analysis guided interpretation, informed by an intersectional lens.

**Findings:**

Five themes described mothers’ experiences: early educational disruption and redirected aspirations; continuous caregiving with uneven support; barriers to maternal healthcare access; emotional distress and psychological strain; and identity transformation. These intersecting challenges contributed to significant mental health strain and highlighted persistent gaps in access to supportive, adolescent-responsive maternal care.

**Conclusions:**

Adolescent mothers navigate motherhood with substantial emotional and practical burdens yet demonstrate meaningful resilience. Findings highlight the need for adolescent‑responsive mental health support, accessible care, routine and confidential distress screening, referral pathways, and supportive educational and community‑based interventions tailored to underserved rural settings.

## Introduction

Adolescent motherhood is a pressing yet underrepresented health and social concern in Pakistan, where cultural, economic, and gendered structures shape reproductive choices and maternal experiences. Despite global commitments to improving adolescent reproductive and mental health, millions of adolescent girls (aged 10–19 years) in Low- and middle-income countries (LMICs) become mothers before adulthood, often with inadequate support [1]. Pakistan has one of the highest rates of adolescent pregnancies in South Asia, with 42 out of every 1,000 girls giving birth before age 19 [2].

The transition to motherhood, from pregnancy to early parenting, is a major life transformation marked by physical, emotional, and social change [3,4]. For adolescents, whose own development is still underway, this transition can be overwhelming. Previous research in low- and middle-income settings shows that adolescent mothers are more likely to experience depression, anxiety, postpartum distress, and suicidal ideation [5,6]. These conditions are often compounded by isolation, stigma, limited decision-making power, and unpaid domestic labor [7,8].

In rural Pakistan, early marriage, poverty, low female literacy, and sociocultural norms that prioritize women’s roles as wives and mothers over education or autonomy heighten vulnerability to early motherhood and psychosocial burdens [9–12]. These challenges are further intensified by rural barriers to care, including long travel distances, transportation limitations, restricted service availability, and dependence on family decision-makers for healthcare access. While substantial literature documents the physical health risks of adolescent pregnancy, such as anemia, eclampsia, and neonatal complications [4,13,14], there remains a significant gap in understanding how adolescent mothers in Pakistan experience the transition to motherhood as an emotional, social, and structurally shaped process. Existing work has often emphasized biomedical outcomes, prevalence estimates, or general risk factors, leaving insufficient attention to how adolescent mothers themselves describe distress, caregiving burden, educational disruption, barriers to care, and changing identity during this transition.

This gap is important for health research and practice because adolescent mothers in underserved settings may face overlapping developmental, reproductive, mental health, and health-system vulnerabilities during a critical period of transition. Yet these needs often remain poorly understood within maternal health systems that primarily focus on pregnancy and childbirth outcomes. By centering adolescent mothers lived experiences, this study contributes qualitative evidence on how mental health strain is produced through everyday caregiving demands, interrupted life trajectories, service inaccessibility, and limited recognition of emotional needs during the transition to motherhood.

This study explored how adolescent mothers in Matiari, Pakistan understands and experience the transition to motherhood, with particular attention to the challenges that affect their mental health during this period.

## Materials and methods

### Research design

This study used a focused ethnographic design to explore how adolescent mothers in Matiari, Sindh, Pakistan, experience the transition to motherhood and the mental health challenges associated with that transition. Focused ethnography is particularly suited to examining a specific issue within a clearly defined group and context, especially when the aim is to understand meanings, practices, and experiences related to a particular phenomenon rather than to describe an entire culture [15,16]. In the present study, the bounded phenomenon was adolescent motherhood in a rural Pakistani setting.

The study was informed by an Meleis’ Transition Theory and intersectional lens [17,18]. Transition theory was relevant because adolescent motherhood involves developmental, relational, social, and health-related transitions, including pregnancy, childbirth, caregiving, and maternal identity formation [18]. It was used to inform the broad areas of inquiry in the semi-structured interview guide. The intersectional lens guided attention to how participants’ experiences were shaped by the intersection of multiple social and structural forces. Analysis remained inductive and grounded in participants’ accounts; themes were developed through close reading, coding, and comparison of interview, observation, field note, and artifact data. Intersectionality was then used to interpret how overlapping identities and contexts shaped participants’ transition to motherhood. This study was conducted and reported with attention to the Consolidated Criteria for Reporting Qualitative Research (COREQ) checklist [19].

### Study setting

The study was conducted in Matiari, predominantly a rural district in Sindh Province with a population of approximately 0.77 million [20]. Matiari is an agrarian area characterized by dispersed village settlements, limited transportation infrastructure, constrained access to formal health services, and gendered sociocultural norms that shape adolescent girls’ educational, marital, and reproductive trajectories.

The district includes three administrative sub-divisions (talukas): Hala, Matiari, and Saeedabad. Female literacy remains low at 30%, and early marriage is common, with approximately 24% of adolescent girls aged 15–19 reported as married [21]. The public health infrastructure in Matiari includes 7 Basic Health Units (BHUs), 3 Rural Health Centers (RHCs), 3 Mother and Child Health Centers (MCHCs), and one District Headquarter Hospital. RHCs and BHUs are mainly in rural areas, while hospitals and MCHCs are in urban centers. However, access to these services is constrained by limited transportation, gender norms restricting women’s mobility, and lack of adolescent-friendly care [22]. About 42% of births occur at home, with a significant proportion attended by Traditional Birth Attendants (TBAs) without formal medical training [23].

### Participants and sampling

Twenty-five adolescent mothers were recruited through purposive and snowball sampling with the support of Lady Health Workers (LHWs), who provide maternal and child health services in rural communities and maintain pregnancy-related records at the community level. Participants were identified based on predefined criteria: women aged 15–19 at the time of pregnancy, currently residing in Matiari, married at the time of pregnancy (pregnancy outside marriage is highly stigmatized in this context and is rarely disclosed), and able to speak Urdu or Sindhi. Participants ages at the time of interview ranged from 18–25 years. There was no maximum age criterion at the time of interview because the study focused on experiences of adolescent transition to motherhood. Purposive sampling was used to recruit participants with direct experience of the phenomenon under study, while snowball sampling enabled the identification of additional eligible mothers through community networks. A member of the research team (Z.M), who worked in the community, introduced the lead researcher (A.H) to the local LHWs. The lead researcher then held multiple meetings with LHWs to discuss the research aims, objectives, recruitment procedures. LHWs were then invited to identify potentially eligible participants and served as the first point of contact. They introduced the study to eligible participants, and shared participants’ details with the lead researcher only after participants agreed. The lead researcher then confirmed eligibility and obtained informed consent directly from participants. To protect confidentiality and reduce perceived influence, LHWs were not present during interviews and did not have access to study data. Participants were recruited between October 2024 and May 2025.

### Data collection

Data were collected through semi-structured in-depth interviews, observations, and artifact elicitation. The interview guide consisted of open-ended questions and follow-up prompts designed to elicit adolescents’ accounts of pregnancy, childbirth, early caregiving, family relationships, access to health care, emotional experiences, and changes in self-perception following motherhood. Lead questions were used consistently across interviews, while probes were adapted iteratively to explore issues raised by participants in greater depth and to clarify emerging patterns in the data. The interview guide was informed by focused ethnographic principles and sensitized by Meleis’ Transition Theory (refer to the supplementary file A). It included open-ended questions about participants’ experiences of change during pregnancy, childbirth, and motherhood; preparation for motherhood; changes in identity and relationships; sources of support; barriers to care; emotional responses; and strategies used to manage maternal responsibilities. The guide was developed in English, translated into Sindhi (local language), reviewed by the research team members familiar with local cultural context for relevance and clarity, and used flexibly, with probes adapted to participants’ responses. The guide was pilot tested with three adolescent mother, and minor revisions were made to improve wording, flow, and for cultural clarity. Pilot data were included in the final analysis.

The use of observations and artifact elicitation provided additional contextual and material insight into the everyday lived experience of motherhood and supported triangulation of findings across data sources. Observations were conducted in everyday community settings, including village streets, local bazaars, agricultural fields, and surrounding public spaces. Observations focused on caregiving practices, social interactions, mobility, fieldwork responsibilities, and the broader social and material conditions shaping everyday motherhood.. Artifacts, including baby clothing, feeding items, religious objects, and household materials, were treated as meaningful elements of maternal life and helped illuminate participants’ experiences of pregnancy, caregiving, emotional attachment, and motherhood.

Interviews (40–60 minutes) were conducted in person (n = 13) or via Zoom (n = 12)**,** by the first author, a female doctoral researcher with training in qualitative methods and prior experience in maternal and community health research. Participants had no prior relationship with the interviewer before recruitment. Interviews were conducted in local language (Sindhi), audio-recorded, and transcribed in the original language. Transcripts were then translated into English. Translation was led by the primary researcher (A.H), who had linguistic and cultural familiarity with Sindhi and English. Back-translation and comparison with original-language transcripts were used to support accuracy and preserve participants’ intended meanings. Field notes and observational notes were maintained during and after data collection to capture contextual details and non-verbal observations where possible, and emerging analytical insights. Data collection and preliminary analysis occurred iteratively and continued until data saturation was reached, with no substantially new patterns emerging in later interviews.

### Data analysis

Data analysis was conducted alongside data collection and followed reflexive thematic analysis, informed by Braun and Clarke’s approach [24]. Analysis proceeded through repeated reading of transcripts, initial coding, development of candidate themes, review and refinement of themes, and definition and naming of the final themes. NVivo 12 software was used to organize the data and support systematic coding and retrieval. Coding was primarily inductive in that themes were developed from participants’ accounts; however, interpretation was also informed by the study’s intersectional lens.

Several strategies were used to strengthen trustworthiness, drawing on Lincoln and Guba’s criteria [25]. Credibility was supported through triangulation across interviews, observations, field notes, and artifacts; iterative engagement with the data; and regular analytic discussions among the research team. Reflexivity was maintained through memo-writing and documentation of the researcher’s positionality, assumptions, and evolving interpretations throughout data collection and analysis, which also supported confirmability. Contextual richness and transferability were supported through detailed description of the study setting, participants, and analytic process. Dependability was strengthened by maintaining an audit trail of coding decisions, theme development, and revisions across stages of analysis.

### Ethical considerations

Ethical approval for the study was obtained from the University of Alberta’s Research Ethics Board (Pro00142593) and National Bioethics Committee, Pakistan (NBC-1128/23/377). After the study was explained, participants were given adequate time to consider participation and ask questions before deciding whether to participate. Written informed consent was obtained from all participants prior to data collection. Consent forms were provided in local language (Sindhi). For participants with limited literacy, the researcher explained the study purpose, procedures, risks and benefits, confidentiality, voluntary participation, and the right to withdraw in simple language. Participants were given time to ask questions and confirm understanding before documenting consent by signature or thumbprint. With participants’ permission, verbal confirmation of consent was also audio-recorded where appropriate. Participation was voluntary, and confidentiality was maintained by removing identifying information and securely storing all data.

## Findings

We conducted in-depth interviews with 25 mothers in Matiari, Sindh, Pakistan, to explore their experiences of adolescent motherhood and the mental health challenges associated with that transition. No eligible participants who were approached declined participation. Participant characteristics are summarized in Table 1. Participants were asked about their husbands’ ages; however, most were unable to recall this information. Therefore, husbands’ ages are not included in Table.

**Table 1:** Characteristics of the study participants.

Characteristics	Number of Participants
**Marital Status**	
Married	24
Married (second marriage)	1
**Currently Studying**	
Yes	1
No	24
**Education**	
No formal education	16
Grade 1–5	5
Grade 6–12	4
**Occupation**	
Housewife	21
Labourer	1
Stitching work	2
Works at an NGO	1
**Husband’s Education**	
No Formal Education	8
Grade 1–5	3
Grade 6–12	12
Undergraduate	1
Graduate	1
**Monthly Family Income**	
Less than 25,000 PKR (**<~USD 90**)	8
25,000 to <50,000 PKR (**~USD 90–180**)	11
50,000 to <75,000 PKR (**~USD 180–270**)	1
75,000 to <100,000 PKR (**~USD 270–360**)	3
100,000 PKR or more (**≥ ~ USD 360**)	2
**Type of Family**	
Extended	20
Nuclear	5
**Age at the time of Interview**	
18 years	3
19 years	10
20 years	2
21 years	5
22 years	2
23 years	1
24 years	1
25 years	1
**Age at Marriage**	
12 Years	1
14 years	4
15 years	9
16 years	6
17 years	3
18 years	1
19 years	1
**Age at First Pregnancy**	
15 years	5
16 years	3
17 years	10
18 years	5
19 years	2
**Number of Children**	
1	16
2	3
3	4
5	2

We developed five interrelated themes: (1) interrupted education and redirected aspirations; (2) burden of continuous caregiving and uneven support; (3) barriers to healthcare access; (4) emotional distress and psychological strain; and (5) identity transformation during adolescent motherhood.

### Interrupted education and redirected aspirations

Participants consistently described the interruption of formal education as one of the earliest and most consequential disruptions in their transition to adolescence and motherhood. School represented routine, friendship, enjoyment, aspiration, and a sense of self.

Several participants described leaving school at the onset of menstruation, often because of stigma and lack of support made continued attendance difficult. One participant recalled, *“When I got my first period, my parents discontinued my schooling because, in our society, girls are not allowed to continue education after they start menstruating.” —P-02.* Menarche appeared to operate as a physical marker that shifted girls toward expected futures of marriage, domestic responsibility, and motherhood. Participants’ sorrow about leaving school reflected the loss of education as well as the narrowing of future possibilities once bodily maturity became linked to adult gendered roles.

For some participants, the consequences of limited education continued into pregnancy and motherhood, affecting confidence and their ability to engage with health information. One mother explained, *“Because I never studied, I couldn’t understand health information during pregnancy. When doctors told me things, I didn’t always understand their language. Even now, when my child grows, I feel bad that I cannot read, write, or guide him in school later. This lack of education makes me feel less confident compared to others” — P-15*. In this way, educational loss remained present in participants’ everyday lives, shaping how they understood themselves as mothers and limiting their sense of competence and authority.

Despite these interruptions, many participants expressed a lasting emotional connection to education. Learning was remembered not only as enjoyable but also as a source of identity and aspiration. The loss of schooling was frequently described as a source of regret, especially when reflecting on missed opportunities for personal growth and future independence. As one mother stated, *“Sometimes I sit and think, ‘If I had studied, maybe my life would be different.’ That regret stays with me.” —P-08.* This regret reflected an ongoing psychological burden linked to unrealized goals, reduced autonomy, and the feeling that an important part of the self had been prematurely closed off.

Following these early disruptions, many adolescent mothers described a shift in how they understood aspiration. Their hopes moved from themselves to their children. Education, dignity, and independence were imagined for the next generation rather than for their own lives. One mother explained this shift directly: *“Now my biggest goal is to educate my child so he can have a better future. Even if my own dreams ended, I want to see my child’s dreams fulfilled.” — P-16*

In this way, interrupted education remained psychologically active in participants’ lives, shaping regret, self-perception, and the meanings they attached to motherhood, care, and their children’s futures.

### Burden of continuous caregiving and uneven support

Adolescent mothers described a sudden shift into full responsibility for infants, husbands, and household work, with little time for rest or personal care. Daily routines were shaped by continuous tasks that began early in the morning and extended late into the night. Many recalled managing childcare and domestic labor at the same time, often while still recovering from childbirth or navigating late pregnancy. One participant captured this shift in identity and responsibility: *“I felt that I was now becoming a mother and needed to think about everything carefully. Before, I was carefree, but now I have to take care of the baby as well as myself.” — P 01.* This workload intensified when infants cried, became ill, or needed constant attention, leaving mothers with little space to meet their own needs. As one mother described, *“From* *morning till night, I’m cooking, cleaning, taking care of my baby and husband. I barely sit down.”* — P 02. These unbroken routines created physical strain and emotional pressure, especially when mothers felt they had no opportunity to pause or recover. Field observations also showed how caregiving continued throughout mothers’ daily routines. Some mothers watched children while working in agricultural fields, showing how childcare was often managed alongside other domestic and fieldwork responsibilities.

Participants described support as central to how they managed caregiving, household work, and emotional strain, yet they emphasized that it was uneven and often unpredictable. Some received meaningful help from husbands, relatives, or neighbors while others described caring alone despite living in large households. One participant explained how her husband’s involvement eased her burden: *“When I’m busy feeding the child, he takes care of his clothes, gets his own food, or does other tasks by himself.” — P‑21.* Another shared, “He used to take me to the doctor and bought whatever I needed. During the delivery, he was right beside me” — P-06. In contrast, others described absent or minimal spousal support: “I am raising my child without any support. My husband never supported me, not even once” — P-20. Lack of support also affected care-seeking: “My daughter got very sick, and my husband said I don’t take care of her properly, so he refused to buy medicines. I went to the public hospital myself, got her checked, and brought medicines for her” — P-14. This pattern of mothers carrying care-seeking responsibilities alone was also visible outside a small government clinic in Matiari, where young mothers waited with babies or small children in their laps while managing prescriptions, medicine bags, breastfeeding, and crying children without visible male or family support.

Support from family also shaped motherhood experiences. Some participants described shared household work: *“If I’m busy with my child, one of them might cook or sweep the floor…* *It’s like teamwork” — P-01*. In contrast, others described moments when support was absent. As one mother shared, *“Even when I cried, they said I was being dramatic. I wanted someone to just sit with me and listen.”— P-21.* These accounts suggest that support functioned as practical assistance as well as an emotional buffer; when absent, caregiving was experienced as both relentless labor and psychological isolation.

### Barriers to healthcare access

Participants described multiple barriers to accessing maternal healthcare, including long travel distances, lack of transportation, financial constraints, and disrespectful treatment within healthcare facilities. These financial, geographic, and systemic barriers often left mothers feeling abandoned and devalued by the very systems meant to protect them.

Long travel distances and transport costs were particularly burdensome. One participant described: *“It takes us almost 3 hours to get there… We booked a car for 2,000 rupees, which was a lot for us.” — P-23.* Field observations similarly showed how care-seeking was shaped by distance and waiting; in one instance, two pregnant women waited for a long time for transport to reach the hospital. Such accounts and observations illustrate how geographic and economic constraints could delay or complicate care-seeking, especially for adolescent mothers with limited mobility and few independent resources.

Even when participants did reach facilities, their experiences of care were often undermined by neglect, poor communication, or disrespect. One mother stated: *“Doctors never explained procedures… They shouted if we asked too many questions. I felt very small, like I had no right to care because I was poor.” — P-15.* Another participant similarly emphasized the lack of guidance within healthcare settings: *“Mostly we had to wait too long at the hospital. The doctors wouldn’t give us proper time. If we didn’t know someone there, we had to wait for* *hours… Sometimes I needed emotional guidance or just someone to explain things in simple words, but no one did that… There’s no one to guide young mothers like us” — P-06.* These encounters had emotional consequences beyond the immediate clinical interaction. Participants described feeling humiliated, frightened, and devalued, suggesting that mistreatment within health services contributed to emotional withdrawal and made care feel less supportive.

### Emotional distress and psychological strain

Across participants’ accounts, adolescent mothers described substantial emotional strain during pregnancy, childbirth, and the postpartum period. Although they did not use clinical terms, their narratives showed persistent fear, anxiety, cognitive overload, irritability, and moments of despair. These experiences were rarely acknowledged by families or health providers, leaving many to manage distress alone.

Fear was a constant presence, shaped by stories of medical complications, past losses, and the unpredictability of childbirth. One participant described this early fear clearly: *“I used to hear stories… This made me worry that I might also have to go through a C section. I feared something might happen to me or my baby.” — P 06.* Economic hardship further intensified emotional burden. As one mother stated: *“Many times we had no money for food. Some nights, I only drank water and put my children to sleep without meals… It is very painful for a mother when she cannot feed her children.” — P-14.* Cognitive strain was also common. Mothers described forgetfulness, confusion, and difficulty concentrating, especially when caring for newborns and managing household tasks. These lapses disrupted daily routines and created frustration and shame. One participant shared, *“I would forget simple things or get confused, especially if there were too many tasks to do at once.” — P 17*

For some participants, emotional overload escalated into thoughts of self‑harm during moments of exhaustion and hopelessness. These thoughts were described as brief but frightening, often countered by a sense of responsibility toward their children. As one mother stated, *“Sometimes I felt so exhausted and hopeless that I even thought about ending my life… But whenever those thoughts came, I reminded myself that I have to live for my children.” — P-15.* These accounts indicate that emotional distress formed a central dimension of how many participants experienced the transition to motherhood.

### Identity transformation during adolescent motherhood

Participants described adolescent motherhood as reshaping how they saw themselves, their bodies, and their place in the world. This transformation involved both loss and growth. Many mothers mourned the abrupt ending of girlhood and the disappearance of a more carefree sense of self, while others described emerging strength, purpose, and voice through caregiving and endurance. Across these accounts, identity transformation was experienced as a gradual and emotionally complex process rather than a simple shift into adulthood.

#### Losing girlhood and living in a changed body.

Participants spoke of adolescent motherhood as an abrupt movement from adolescence into adult responsibility. Several described feeling that a younger version of themselves had been left behind too quickly. One participant captured this contrast by comparing her life with friends: *“I could see my life was different from my friends who were still unmarried and carefree. I was already running a household and thinking about a child’s needs. Still, deep inside, I knew my childhood had ended earlier than theirs.” — P-03.* Another described the internal change that followed this transition: *“I feel like I’ve become someone else.” — P-24.* Another mother described this compressed transition more directly: *“I feel like a different person now. Sometimes* *I feel… like I’ve lost who I was. I don’t laugh or joke like before. I just think about my child all day.” — P-11.* These accounts reflected more than role change; they conveyed a sense of developmental loss. Maternal identity was therefore adopted over time and absorbed within social expectations that required participants to become responsible wives and mothers while they were still negotiating their own growth.

Participants also linked these changes to the body. Fatigue, pain, and visible bodily changes altered confidence and contributed to a sense of premature aging. One mother reflected: *“Sometimes I looked at myself in the mirror and thought, ‘You are only 17, but you already look tired and old.’ —P-16* Another described feeling erased from the world of girlhood: *“I used to feel good about myself before, but not anymore… now I feel like I’ve faded.” — P-20.* For some, bodily change also marked a difference from other girls their age. One participant reflected, *“Now, when I see those marks, I think of them as signs of my journey. But still, sometimes when other girls talk about beauty or clothes, I feel like I’m not part of that world anymore.” — P-17.* In these narratives, bodily change became part of a broader experience of losing girlhood and adapting to a new, often unfamiliar sense of self.

#### Strength, meaning, and an emerging voice.

At the same time, participants described forms of resilience that emerged through caregiving, faith, emotional endurance, and reflection. Some mothers expressed pride in their capacity to keep going despite strain. One participant stated: *“I feel strong when I see that I’ve done everything for my child on my own*.” *— P-02*. Another described how her child’s affection grounded her during stress: *“When my kids hug me or call me ‘Amma’ (means mom), my heart melts… These moments give me strength.” — P-16.* This strength often emerged through the everyday demands of caregiving and the gradual realization that they were capable of enduring more than they had expected. One participant explained*, “Every stage taught me something about myself—how patient I can be, how strong I am.” — P-01.* These accounts suggest that resilience developed through hardship and everyday caregiving.

Some participants also described an emerging voice shaped by experience. They offered advice to younger girls, challenged assumptions that girls exist only for marriage and motherhood, and imagined support structures they did not have. One participant stated this need directly: *“We need safe spaces for girls like me to talk, learn, and be heard openly.” — P-22.* Another emphasized the importance of broader possibility: *“Girls are not just made for marriage and motherhood; they can do so much more if they’re given the opportunity.” — P-06.* In this way, identity transformation involved loss, meaning-making, new forms of self-understanding, and the emergence of a stronger voice.

## Discussion

This focused ethnographic study explored how adolescent mothers in rural Pakistan experienced the transition to motherhood and the mental health challenges associated with that transition. Participants described motherhood as a period marked by educational disruption, intense caregiving demands, uneven support, barriers to healthcare access, emotional distress, and changes in identity. Taken together, these findings suggest that mental health strain among adolescent mothers emerged through the intersection of developmental challenges, gendered expectations, economic hardship, and limitations in maternal health services. Participants also described resilience, meaning-making, and emerging strength that developed within conditions of considerable constraint and limited support.

The study contributes to the literature on adolescent motherhood in three ways. First, it addresses a gap in prior work by centering adolescent mothers’ own narratives of psychological and emotional experience during the transition to motherhood in a rural Pakistani setting. Second, it extends research that often treats adolescent pregnancy primarily as a biomedical or demographic issue by showing how mental health strain is embedded in everyday caregiving, constrained mobility, educational disruption, and encounters with health services. Third, by applying an intersectional lens, the study demonstrates how age, gender, poverty, and marital status converge to shape vulnerability, recognition, support, and lived experience during early motherhood.

Educational interruption was among the earliest disruptions and carried lasting emotional consequences. Consistent with previous studies, participants were withdrawn from school at puberty, based on beliefs that education conflicts with a girl’s roles as wife and mother [26,27]. In this context, menarche marked a shift in how participants were treated, narrowing educational possibilities and increasing emotional awareness of future caregiving expectations. Previous research shows that early school dropout contributes to diminished self‑esteem, social isolation, and long‑term regret [28]. This was reflected in participants’ accounts of schooling as a source of identity and aspiration. The emotional loss associated with discontinued education often resurfaced in adulthood, shaping feelings of missed opportunity and reduced autonomy. Redirecting aspirations toward their children functioned as both a coping mechanism and a way to manage grief over their own interrupted futures, echoing patterns documented in prior studies [29,30].

The transition into motherhood brought an abrupt immersion into continuous caregiving and domestic labor, intensifying emotional strain. Participants described long, uninterrupted days of childcare and housework, often while still recovering physically. These expectations align with research on intensive mothering, where adolescent mothers are expected to meet all caregiving needs through self‑sacrifice [31]. The physical exhaustion of unpaid labor was closely tied to irritability, anxiety, and emotional overload, consistent with previous findings on adolescent maternal stress [32]. While some mothers found meaning in caregiving, many experienced inconsistent or absent support, which heightened feelings of loneliness and emotional invalidation [33]. In line with broader theoretical work on role change and adjustment, the availability of practical and emotional support shaped participants’ confidence, coping, and sense of connection during early motherhood [34] Husbands’ involvement or absence influenced how participants experienced caregiving and psychological strain. Importantly, support functioned as practical assistance as well as a psychological buffer; when absent, adolescent mothers appeared to experience caregiving as both labor and emotional isolation.

From an intersectional perspective, this emotional neglect reflects how gender and age intersect to shape adolescent mothers’ experiences when they seek support [17,35]. Participants also described significant challenges accessing healthcare. Long travel distances, high costs, and disrespectful treatment created fear, anxiety, and feelings of worthlessness. These experiences mirror global evidence that young, poor women often receive inadequate or judgmental care [36,37]. Even when mothers overcame logistical barriers, mistreatment left them feeling undeserving of help, reinforcing emotional withdrawal, and reducing help‑seeking. Previous research similarly shows that disrespectful maternity care increases fear and avoidance of services among adolescent mothers [38]. For adolescent mothers, access barriers were therefore geographic, financial, and emotional, shaping whether care felt reachable, safe, and worth pursuing. The findings suggest that disrespectful care not only limits service use but also deepens emotional distress, reinforcing cycles of avoidance [17].

Emotional distress was a cross-cutting theme. Participants described persistent fear, sadness, cognitive overload, and moments of despair, symptoms consistent with depression and anxiety among adolescent mothers [39]. Economic hardship intensified distress, particularly when mothers struggled to meet basic needs. Cognitive strain, including forgetfulness and difficulty concentrating, reflected the mental load of managing multiple responsibilities with little rest. These findings align with literature showing that adolescent mothers often mask emotional suffering due to stigma, leading to under‑recognized mental‑health needs [40]. Importantly, participants rarely framed these experiences in clinical language, suggesting that maternal distress may remain invisible within households and health systems when suffering is normalized, minimized, or interpreted only through practical hardship. Finally, adolescent motherhood reshaped participants’ sense of self in ways that carried both emotional loss and psychological growth. Many mourned the abrupt end of girlhood and described feeling prematurely aged or diminished, consistent with research on disrupted developmental trajectories [41]. Yet participants also demonstrated resilience through caregiving competence, faith, and emerging voice. These forms of strength reflect feminist scholarship that frames resilience as daily survival within constraint [42]. These findings indicate that resilience among adolescent mothers is context‑dependent, relational, and shaped by intersecting social pressures rather than individual traits alone.

Limitations include the study’s focus on one district, which may limit transferability, and reliance on self‑reported experiences, which may be influenced by recall or social desirability. In addition, because participants were recruited from a specific sociocultural and geographic context, the findings may not reflect the experiences of all adolescent mothers in other rural or urban settings in Pakistan. However, triangulation across interviews, observations, and artifacts strengthens the credibility of the findings.

### Implications for practice and policy

These findings have several implications for maternal and adolescent health practice and policy, particularly in strengthening health systems to better recognize and respond to adolescent mothers’ mental health needs. First, maternal care providers, including Lady Health Workers and community midwives, should be better equipped to recognize and respond to the physical and emotional needs of adolescent mothers, particularly when signs of distress may otherwise go unnoticed or be minimized. Training for frontline maternal health providers should include adolescent-responsive communication, basic mental health awareness, and strategies for identifying distress in contexts where emotional suffering may be expressed indirectly through physical complaints, silence, or withdrawal.

Second, routine perinatal mental health screening and referral pathways should be integrated into antenatal and postnatal, and child health services in ways that are feasible, developmentally appropriate, and accessible for adolescents. Brief screening approaches could include locally adapted versions of the Edinburgh Postnatal Depression Scale, the Patient Health Questionnaire, or short distress-screening checklists administered by trained LHWs, community midwives, or primary care providers. Screening should be accompanied by supportive conversation, privacy, follow-up, and clear referral pathways for adolescents who disclose severe distress, self-harm thoughts immediate safety concerns, or psychosocial needs.

Third, because educational disruption emerged as a major source of emotional loss and reduced future opportunity, community- and policy-level efforts are needed to support continued education after marriage or childbirth. Community-based initiatives could include mother–adolescent support groups, safe spaces for married and parenting girls, home-based or flexible learning programs, links with local schools or non-formal education providers, literacy support, and community dialogues with parents, husbands, mothers-in-law, religious leaders, teachers, and health workers. These initiatives should address the belief that menstruation, marriage, or motherhood marks the end of girls’ educational possibilities. The findings also suggest a need for stronger health outreach strategies, including transportation-sensitive maternal care planning, mobile or community-based service delivery, and safe, nonjudgmental spaces where adolescent mothers can receive emotional support, share experiences, and build practical life and parenting skills. Peer-support models, home-visiting approaches, and locally embedded maternal support initiatives may be particularly useful in settings where adolescent mothers face mobility restrictions or limited autonomy in seeking care. Future research may also examine pregnancy intention and contraceptive use to better understand the reproductive circumstances surrounding adolescent mothers’ transition to motherhood.

Finally, adolescent mothers should be included more meaningfully in the design of reproductive, maternal, and social support services so that interventions are informed by the realities of their lived experiences rather than assumptions about their needs.

## Conclusions

This study provides an in-depth understanding of the lives of adolescent mothers in a Pakistani context and highlights how the transition to motherhood during adolescence carries significant emotional and psychological challenges. Participants’ narratives reveal persistent mental health strain shaped by early educational disruption, heavy caregiving responsibilities, barriers to healthcare access, and limited recognition of adolescents’ emotional needs during pregnancy and adolescent motherhood. These findings suggest that adolescent mothers’ distress is not solely an individual experience but is closely tied to the structural and social conditions that shape motherhood in underserved settings.

These findings underscore the importance of recognizing adolescent mothers as a distinct group within maternal health systems whose mental health needs often remain invisible within routine care. Strengthening adolescent-responsive maternal healthcare, integrating mental health screening into perinatal services, and expanding supportive programs that enable continued education and social support are critical for improving well-being during the transition to motherhood. Such efforts are particularly important in rural settings, where geographic, social, and service-related barriers may further limit adolescent mothers’ access to timely and supportive care. Despite considerable challenges, many participants demonstrated resilience through caregiving, reflection, and hope for their children’s futures. Supporting adolescent mothers through responsive healthcare services, community-based support mechanisms, and policies that address the well-being of adolescent mothers can contribute to healthier developmental trajectories for both mothers and their children. Although situated in Pakistan, these findings contribute to broader health scholarship by illustrating how structural inequities, service inaccessibility, and unmet psychosocial needs shape adolescent motherhood across underserved settings.

## Supporting information

S1 File Supplementary file A. Interview guide used for semi-structured interviews.
(DOCX)

S2 File Inclusivity in global research questionnaire. Completed PLOS Inclusivity in Global Research Questionnaire.
(DOCX)
