# Supplementary material for: Understanding the transition to motherhood among adolescent mothers in rural Pakistan: Implications for mental health
Source: PLoS One. 2026 Jul 30;21(7):e0355175. doi: 10.1371/journal.pone.0355175 (PMC13422825; doi:10.1371/journal.pone.0355175)
Supplement: S1 File — Interview guide used for semi-structured interviews. (DOCX) [file pone.0355175.s001.docx]

**Semi-Structured Interview Guide**

| **Interview Guide Questions** | **How Meleis’ Transition Theory Sensitized the Interview Area** |
| --- | --- |
| - Can you tell me about yourself? - Share your experiences before you became a mother. | Provides contextual understanding of participants’ lives before motherhood. This section was not directly tied to one specific concept but helped situate the transition experience. |
| - Can you share your experience when you first found out you were pregnant? | Helped frame the beginning of the transition, including early awareness, meaning, and emotional response. |
| - Provide a detailed journey through each stage as you transitioned into motherhood, from pregnancy to till the first year of child birth. | Oriented the guide toward the transition process over time, from pregnancy to childbirth and early motherhood. |
| - Describe your experiences after becoming a mother. - How independent are you with your role of being mother? | Informed exploration of change, adjustment, role engagement, and independence in the maternal role. |
| - Can you share the challenges encountered during the transition into motherhood. | Helped explore conditions that made the transition easier or more difficult. |
| - Reflect on the impact of motherhood on your mental health. - How did your mental health influence your interactions with your child? | Informed exploration of emotional well-being and its influence on the motherhood experience. |
| - Share how you managed the challenges you faced? - What support you received during pregnancy and motherhood? - With whom do you discuss your challenges and experience? - Share what resources or services that you found particularly helpful or lacking during your journey into motherhood. | Helped frame questions about coping, support, connectedness, and available or missing resources. |
| - What did you learn during your time as an adolescent mother that you think would help others in similar situation? - What advice would you give to people who can support adolescent mothers? - What suggestions do you have for the government to better support adolescent mothers? - How can healthcare be better for young mothers? Any suggestions for improvement? - Looking back, is there anything you wish you had known or done differently? | Informed exploration of lessons learned and possible supports, services, and interventions for adolescent mothers. |
| - Is there anything else you would like to share about your journey into motherhood that we haven’t discussed? | Allowed participants to share additional experiences beyond the main interview areas. |
